# Ensuring Global Food Security by Improving Protein Content in Major Grain Legumes Using Breeding and ‘Omics’ Tools

**DOI:** 10.3390/ijms23147710

**Published:** 2022-07-12

**Authors:** Uday C. Jha, Harsh Nayyar, Swarup K. Parida, Rupesh Deshmukh, Eric J. B. von Wettberg, Kadambot H. M. Siddique

**Affiliations:** 1 ICAR—Indian Institute of Pulses Research (IIPR), Kanpur 208024, India; 2 Department of Botany, Panjab University, Chandigarh 160014, India; harshnayyar@hotmail.com; 3 National Institute of Plant Genome Research, New Delhi 110067, India; swarup@nipgr.ac.in; 4 National Agri-Food Biotechnology Institute, Punjab 140308, India; rupesh@nabi.res.in; 5 Department of Plant and Soil Science, The University of Vermont, Burlington, VT 05405, USA; eric.bishop-von-wettberg@uvm.edu; 6 The UWA Institute of Agriculture, The University of Western Australia, Perth, WA 6001, Australia

**Keywords:** grain legume, protein, biofortification, molecular marker, QTL

## Abstract

Grain legumes are a rich source of dietary protein for millions of people globally and thus a key driver for securing global food security. Legume plant-based ‘dietary protein’ biofortification is an economic strategy for alleviating the menace of rising malnutrition-related problems and hidden hunger. Malnutrition from protein deficiency is predominant in human populations with an insufficient daily intake of animal protein/dietary protein due to economic limitations, especially in developing countries. Therefore, enhancing grain legume protein content will help eradicate protein-related malnutrition problems in low-income and underprivileged countries. Here, we review the exploitable genetic variability for grain protein content in various major grain legumes for improving the protein content of high-yielding, low-protein genotypes. We highlight classical genetics-based inheritance of protein content in various legumes and discuss advances in molecular marker technology that have enabled us to underpin various quantitative trait loci controlling seed protein content (SPC) in biparental-based mapping populations and genome-wide association studies. We also review the progress of functional genomics in deciphering the underlying candidate gene(s) controlling SPC in various grain legumes and the role of proteomics and metabolomics in shedding light on the accumulation of various novel proteins and metabolites in high-protein legume genotypes. Lastly, we detail the scope of genomic selection, high-throughput phenotyping, emerging genome editing tools, and speed breeding protocols for enhancing SPC in grain legumes to achieve legume-based dietary protein security and thus reduce the global hunger risk.

## 1. Introduction

Alarming trends of anthropogenic climate change and environmental deterioration jeopardize global crop yields, resource distribution, and ecosystems, resulting in global food insecurity and undernourishment in the growing human population [1]. An estimated 840 million people globally will be undernourished by 2030 [2]. The COVID-19 pandemic will have compounded this figure, increasing the food-related hunger crisis. Dietary protein is an essential macronutrient for human growth and development, with infants requiring 1.52 g per kg body weight per day and adults recommended 0.80 g per kg body weight per day [3]. Apart from micronutrient deficiency, malnutrition from dietary protein deficiency causes ‘marasmus’, ‘kwashiorkor’ anemia, impaired immunity, and ‘environmental enteric dysfunction,’ most prevalent in developing and low-income countries, especially southern Asia and sub-Saharan Africa [4,5,6]. Most of the people residing in these regions predominantly consume maize, sorghum, and cassava in their daily diets, which are rich in starch but insufficient in protein [6,7]. Thus, many people, especially infants, inhabiting these regions do not consume the required daily protein, affecting their overall growth and development [5,6]. Notably, Europe imports 70% of the plant-based protein consumed by its human population [8], a trend that the increasing global human population will further exacerbate.

Breeding crops, especially legumes, with high-quality traits such as SPC is a promising approach for overcoming these challenges. Grain legumes are one of the richest sources of plant-based dietary protein, providing essential amino acids and supplying the increasing demand for protein-based human diets [9]. Grain legume seeds, popularly known as ‘poor man’s meat’, are the cheapest protein source [10,11,12]. In addition, legume-based protein could be instrumental in minimizing greenhouse gas emissions, helping to protect the environment [13]. Screening genetic variability for protein content in various legume germplasm and crop wild relatives is the first step to identifying high-protein grain legumes for the development of high-yielding, high-protein legumes. A classical genetics-based approach could identify the inheritance pattern of high-protein gene(s) in various legumes. Advances in genomics have enabled the dissection of the genetic architecture of QTLs/gene(s) in various legumes through biparental mapping and genome-wide association studies. Moreover, the availability of complete reference genome assemblies and pangenomes of various legumes could assist in underpinning high-protein genomic regions at the individual or species level. Likewise, advances in functional genomics have enabled the discovery of various candidate genes that improve legume protein content and their precise function. Proteomics and metabolomics can improve our understanding of various complex pathways, molecular networks, and metabolites underlying high-protein grain legumes. Non-destructive phenomics approaches could be instrumental for screening and identifying high-protein lines with high efficiency. Emerging technologies such as genomic selection, rapid generation advancement, and genome editing could be harnessed to improve SPC, eradicate malnutrition related to dietary protein deficiency, and meet the United Nations Sustainable Developmental Goal 2.

## 2. Grain Legumes as an Important Source of Dietary Protein

Grain legumes vary in their protein content, due to fundamental limitations on the components a seed must contain to be viable. Many grains legumes have 25–40% SPC, and it may be difficult to raise that number much beyond 40%. (See Table 1).

Chickpea (*Cicer erietinum* L.) SPC ranges from 17 to 22% before dehulling and 25.3 to 28.9% after dehulling [14,15] (see Table 1). Chickpea seed contains two main proteins—globulin (11S legumin and 7S vicilin) and albumin—with low amounts of glutelins and prolamine; however, the seed is deficient in cysteine and methionine amino acids [43,44]. Despite rich sources of various essential amino acids, cysteine, methionine, valine, and threonine are the major limiting amino acids in chickpea [45]. Desi type chickpea has higher SPC than the kabuli type but no differences in essential amino acids [45].

Common bean (*Phaseolous vulgaris* L.) SPC ranges from 20 to 30% [25,26], and plays a pivotal role in mitigating protein-related malnutrition, especially in underdeveloped countries [46,47]. The major storage protein in common bean is phaseolin, accounting for 36–46% of total seed proteins [48], with 50–60% of the phaseolin belonging to the 7S vicilin class, insufficient in methionine, cysteine, and tryptophan essential amino acids [49,50,51].

Cowpea (*Vigna unguiculata* L. Walp.) is a ‘multi-functional’ grain legume widely used for human consumption. It helps mitigate the challenges of malnutrition in sub-Saharan Africa, and tropical and sub-tropical regions globally [52,53]. Cowpea SPC ranges from 15 to 25% [33,34] (see Table 1). Cowpea storage proteins are abundant in lysine and tryptophan but deficient in methionine and cysteine [53]. Globulins are the most abundant storage protein fraction of cowpea grain, followed by albumins, glutelins, and prolamin [54].

Faba bean (*Vicia faba* L.) SPC ranges from 26 to 41% [30,31,55,56], with abundant essential amino acids except for tryptophan, cysteine, and methionine [57]. More than 80% of the seed proteins comprise globulins (vicilin and legumin) [55]. Of the essential amino acids, faba bean seed is highest in lysine [58].

Lentil (*Lens culinaris* Medik) SPC ranges from 20 to 30% [59]. Like other legumes, lentil seed has a high globulin content (44–70% of storage protein, constituting 11S legumin and 7S vicilin and convicilin) and albumin (26–61% of lentil proteins) but low prolamin and glutelin levels [60,61].

White lupin (*Lupinus albus* L.) seeds are a rich reservoir of protein containing up to 44% [19,62], with two major classes of protein—albumin (15%) and globulin (85%) [63]. The globulin protein comprises α-, β-, γ-, and δ-conglutins [20]. Despite some allergenic effects in white lupin seed protein, they are low in antinutritive properties compared with other grain legumes such as pea and soybean [62,64]. Moreover, white lupin seed contains higher amounts of some important amino acids (lysine, phenylalanine, arginine, and leucine) than soybean, rendering it a high-demand grain legume from a nutritional point of view [65].

Soybean (*Glycine max* (L.) Merr.) is rich in protein, ranging from 35 to 45%. It is deficient in methionine [22,23] but has sufficient lysine to overcome the lysine deficiency of cereals [66]. In 2018, it was estimated that soybean alone contributed 70% of the global protein meal [67].

Mung bean (*Vigna radiata* L.) contains easily digestible protein and several essential micronutrients [68]. It is an excellent source of protein except for sulfur-containing amino acids (methionine and cysteine) [69]. Due to its ease of digestibility relative to other legumes [70] and low hypoallergic properties, mung bean is used as a weaning food for infants [71]. Moreover, mungbean is a good meat substitute for vegetarians and those who cannot afford animal-based dietary protein [12].

Pea (*Pisum sativum* L.) is rich in protein, ranging from 13.7 to 30.7% [37]. Pea seed protein comprises legumin, vicilin, convicilin, and globulin-related proteins [37]. Vicilin is the most abundant protein (26.3–52.0% of total pea protein extract) [37]. Moreover, pea protein is in high demand in food industries due to its gluten-free quality and low allergenicity [72].

Pigeon pea (*Cajanus cajan* (L.) Millsp) seeds contain 20–22% protein and play an essential role in providing plant-based dietary protein to the vegetarian population in India, thus ensuring protein-based food security [73].

Urd bean (*Vigna mungo* L. Hepper) is another important grain legume rich in protein (up to 25%), comprising globulin (63%), albumin (12%), and glutelin (21%) [74]. Urd bean seeds are rich in glutamic acid, aspartic acid, and lysine but deficient in methionine and cysteine [74].

## 3. Harnessing Genetic Variability for Improving Seed Protein Content in Grain Legumes

Harnessing crop germplasm diversity is an economical way to improve important breeding traits, including SPC in grain legume crops [75,76,77,78,79]. Crop genetic resources are the key reservoir for exploring high-SPC genotypes in grain legumes. Considerable amounts of genetic variability for SPC have been captured in chickpea [78,80,81], such as 12.4–31.5% [82], 17–22% [83], and 14.6–23.2% [84]. Serrano et al. [84] identified several high SPC genotypes (LEGCA608, LEGCA609, LEGCA614, LEGCA619, LEGCA716) that could be used to improve chickpea SPC in elite cultivars.

Cowpea is a cheap source of protein for improving human nutrition. Boukar et al. [77] assessed a set of 1541 cowpea lines for genetic variability in grain protein content and mineral profiles. They reported a wide range of genetic variability for SPC (17.5–32.5%), including TVu-2508 (32.2%) [77]. Likewise, Weng et al. [85] screened 173 cowpea accessions collected from various parts of the world at two locations (Fayetteville and Alma, Arkansas). They also reported a substantial amount of genetic variability for SPC (22.8–28.9%), including PI 662992 (28.9%), PI 601085 (28.5%), PI 255765 (28.4%), PI 255774 (28.4%), and PI 666253 (28.4%) [85], which could be used to transfer the high SPC trait into high-yielding elite cowpea varieties. The nutritional profiles (including grain protein content) of 22 cowpea genotypes collected from various regions of eastern, southern, and western Africa were evaluated at two locations in South Africa [34]. Seed protein contents, measured using the combustion method, ranged from 23.16 to 28.13% [34]. The authors noted significant positive correlations between SPC and various mineral contents, indicating the possibility of simultaneously selecting these traits. Among the tested genotypes, 98K-5301 had high Ca and SPC [34]. Similarly, an evaluation of 21 cowpea genotypes identified high SPC in COVU-702 (27.7%) and HC-98-64 (27.9%) [86]. In another study, GonÇalves et al. [87] identified high SPC in Paulistinha (29.2%) among 18 tested cowpea genotypes. An evaluation of 30 Brazilian cowpea lines for protein, vitamin, and mineral content identified high SPC in MNC01-649F-2 (28.3%), BRS-Cauamé (27.8%), BRS-Paraguacu (27.7%), BRS-Marataoa (27.4%), Canapuzinho (25.0%), BRS-Tumucumaque (24.8%), and MNC01-631F-15 (24.6%) [88].

The SPC of selected common bean landraces ranged from 16.54 to 25.23%, while selected modern common bean cultivars ranged from 19.70 to 24.30% (Celmeli et al. [79]; see Table 2).

Grasspea is an inherent climate-resilient grain legume with an excellent source of SPC. An evaluation of 37 grasspea genotypes identified IC127616 rich in SPC (32.2%) [95].

An analysis of 27 local mung bean landraces using the micro-Kjeldahl method identified significant genetic variability for SPC (17.2–29.9%), with the highest values in MGG30 (29.9%), NAGPURI (29.3%), and BSN1 (27.8%) [97]. Moreover, significant genetic variability for SPC (15.2–21%) rich in lysine, tryptophan, valine, leucine, isoleucine and phenylalanine amino acids was noted in *Vigna radiata var sublobata*, a wild species of mung bean [113].

Genetic variability for SPC in lentil ranges from 20 to 30% [76,114,115,116,117]. Likewise, lentil crop wild relatives (CWRs) have significant genetic variability for SPC, such as *L. orientalis* (18.3–27.75%) and *L. ervoides* (18.9–32.7%) [96], which could be used in breeding programs to improve SPC in elite lentil cultivars.

Pea has considerable genetic variability for SPC (19.3–25.2%) (Gottschalk et al. [75]; see Table 2). Wang and Daun [82] reported a SPC ranging from 201.6 to 266.6 g kg^−1^ DM in four elite pea cultivars. In large sets of pea accessions, the SPC was 22–32% [98] and 23–32% [118] using the Kjeldahl technique and 20.6–27.3% [119], 18.6–27.3% [120], 17–27% [121], 17.5–27.8% [122], and 19.3–30.3% [123] using the near-infrared technique. Several promising pea genotypes with high SPC have been identified: CDC Striker (up to 27.8%) [124,125,126,127], Ballet (up to 25.9%) [119,128], Solara (28.8%) [129], Caméor (29.9%), VavD265 (27.5%), and China (32%) [128].

Breeding for high SPC in soybean is a primary objective in soybean breeding programs; however, progress has been limited by the negative relationship between SPC and grain yield and oil content [24,130]. For example, Bandillo et al. [131] and Warrington et al. [132] reported a highly negative correlation between the soybean SPC allele and seed oil content, reducing oil content by 1% for every 2% increase in SPC.

High-protein soybean lines include Danbaegkong (48.9%) [133] and Kwangankong (44.7%) [134], and TN11-5102 selected from 5601T cultivar (421 g kg^−1^ protein on a dry weight basis) [108]. Apart from cultivated species, soybean CWRs (e.g., *Glycine soja*) are an important source of high-protein QTLs [135,136,137]. A population developed by incorporating exotic soybean germplasm exhibited significant genetic variability for SPC [138]. Wehrmann et al. [139] and Wilcox and Cavins [140] backcrossed the high-protein trait from Pando into Cutler 71, a high-yielding low-protein genotype soybean. Later, Cober and Voldeng [101] attempted to transfer the high-protein trait from AC Proteus to Maple Glen; however, the selected progenies exhibited higher protein content than Maple Glen but no yield advantage. Sathia, Seti, Kavre, and Soida Chiny soybean cultivars, collected in Nepal, had high SPC (up to 42–45%) compared to William 82 (39%) and higher arginine (5–10%) content than William 82 (7.4%) [141].

Hence, harnessing the available genetic variability for SPC requires the large-scale screening of land races, CWRs, and grain legume germplasm locked in gene banks across the globe.

## 4. Mendelian Inheritance of Seed Protein Content in Legumes

Several researchers have worked out the genetics of SPC based on Mendelian genetics in various grain legumes [142,143,144]. Considering pea storage proteins (legumin and convicilin), Matta and Gatehouse [145] mapped the *legumin* gene (*Lg-1*), behaving as a single Mendelian gene with five alleles on LG7, and the *convicilin* gene (*Cvc*), behaving as a single Mendelian gene on LG2 using seeds developed from 1238 × 1263, 110 × 807 and 110 × 851 F_2_ crosses. Subsequently, Mahmoud and Gatehouse [146] explained the monogenic inheritance of another pea SPC vicilin (*Vc-1*) gene controlled by two codominant genes located on LG7 using an F_2_ cross from 360 × 611.

Perez et al. [147] revealed the genetic basis of high and low SPC in pea using the genetics of seed size (round vs. wrinkled). They found that round-seeded pea plants (*RR*/*RbRb*) had low SPC with low albumin content, while those with recessive alleles (*rr*/*rbrb*) had high SPC and high albumin content [147]. High heritability of protein content and its control by a few gene(s) is an opportunity to improve protein content in cowpea [92]. Moreover, diallel crosses of six populations derived from two high-protein lines and two high-yielding soybean lines revealed a significant negative correlation between protein content and yield in the high protein × high protein population but a significant positive correlation between protein content and yield in the high yielding × high yielding population [148]. In pigeon pea, an analysis of F_1_ and F_2_ progenies derived from crosses involving four parents revealed a minimum of 3–4 genes controlling protein content [149]. The authors concluded that the low protein trait is partially dominant over the high protein trait.

Various studies have reported a significant effect of environment on SPC [150,151,152]. In soybean, this significant effect involved multiple genes and the quantitative nature of the SPC trait [150,151]. In chickpea, an F_2_ segregating population developed from ICC5912 (blue flowered) × ICC17109 (white flowered) revealed the quantitative nature of the SPC trait and its high negative correlation with seed yield and seed size [78]. A 5 × 5 half diallel cross of cowpea lines revealed the presence of additive and non-additive gene effects for SPC. High seed albumin, prolamin, and globulin were associated with positive effects of the dominant gene, while high SPC and glutelin content were associated with recessive genes [153]. In lentil, Kumar et al. [154] also reported the quantitative nature of the SPC trait. High genetic variation in lentil seed storage protein resulted from high G × E interactions exhibiting moderate heritability (31.3%) [152].

## 5. QTL Mapping for Seed Protein Content

Advances in grain legume genomics have facilitated the identification of underlying QTLs controlling SPC using biparental mapping populations in various grain legumes [118,119,155,156,157].

Few studies have uncovered QTLs controlling SPC in chickpea. However, one study that phenotyped recombinant inbred lines (RILs) derived from ICC995 × ICC5912 across four environments and used a genotyping by sequencing approach delineated one major effect QTL *q-3.2* for SPC that explained 44.3% of the phenotypic variation (PV) on LG3 [158].

In pea, using an F_2_-derived Wt10245 × Wt11238 mapping population, Irzykowska and Wolko [159] mapped five QTLs governing SPC on LG2, LG5, and LG7, explaining 13.1–25.8% PV. Subsequently, two F_5_ mapping populations developed from Wt11238 × Wt3557 and Wt10245 × Wt11238 revealed a QTL for protein content on LGVb flanked by *cp*, *gp*, and *te* markers [118]. Likewise, genotyping an Orb × CDC Striker RIL mapping population with SNP markers identified two SPC QTLs on LG1b, explaining 16% PV, and two on LG4a, explaining 10.2% PV, and genotyping a Carerra × CDC Striker RIL-based mapping population identified four SPC QTLs on LG7b, explaining 13% PV, and one on LG3b [160].

An evaluation of a Terese × K586 RIL population in five different environments identified 14 SPC QTLs located on LGI, LGIII, LGIV, LGV, LGVI, and LGVII [119]. The study identified the underlying candidate gene for the QTL on LGI as the *Rgp* gene (cell wall synthesis) and two underlying candidate genes for the QTL on LGV as *Ls* (GA biosynthesis) and *Rbcs4* (encoding small Rubisco subunit) [119].

Obala et al. [157] gained insight into the genetic determinants controlling SPC in pigeonpea based on the results obtained from five F_2_ populations segregating for SPC (ICP 11605 × ICP 14209, ICP 8863 × ICP 11605, HPL 24 × ICP 11605, ICP 8863 × ICPL 87119, and ICP 5529 × ICP 11605). Fourteen major effect QTLs explaining 23.5% PV were found to be located on CcLG02, CcLG03, CcLG06 and CcLG11 [157].

In soybean, the SPC trait is controlled by multiple alleles and highly influenced by G × E interactions [150]. More than 300 QTLs contributing to SPC in soybean have been reported (http://www.soybase.org, (accessed on 10 May 2022)); [161] and reside across all chromosomes; however, major SPC QTLs are on chromosomes 5, 15, and 20. Diers et al. [155] first reported a major QTL governing high SPC on chromosome 20 in a population developed from crossing cultivated and wild soybean, which was later mapped to a 3 cM on LGI (Nichols et al., 2006) [156]. The location of this QTL was subsequently narrowed to 8.4 Mb [162], <1 MB [163], 77.4 kb [137], and even with only three candidate genes [131] on LG20. Likewise, another major SPC QTL, *qSeedPro_15*, was narrowed to 4 Mb (Zhang et al. [164]; see Table 3), overlapping the previously identified genomic region on chromosome 15 [24,131,155,165,166]. Zhang et al. [164] elucidated a possible candidate gene *Glyma.15G049200* underlying the QTL. Genotyping recombinant inbred lines derived from the interspecific cross of Williams 82 × *G. soja* (PI 483460B) using Illumina Infinium BeadChip sequencing platform identified five SPC QTLs, mapped on chromosomes 6, 8, 13, 19, and 20, explaining 4.6–19.6% PV [167]. Of these identified QTLs, *qPro_20 QTL* was stable across the four tested environments.

SSR, DArT, and DArTseq analysis of five RIL-based mapping populations for high and low SPC and one high × high SPC identified two major QTLs controlling SPC on LG15 and LG20 in soybean [168]. Furthermore, bulk segregation analysis of four high × low SPC mapping populations unveiled novel SPC-controlling genomic regions on LG1, 8, 9, 14, 16, 17, 19, and 20 [168]. An assessment of soybean RILs developed from Linhefenqingdou × Meng 8206 in six different environments identified 25 SPC QTLs explaining up to 26.2% PV [169]. Of the identified QTLs, *qPro-7-1* was highly stable across all tested environments. Recently, Fliege et al. [137] cloned a major SPC governing QTL (*cqSeed protein-003*) and elucidated the underlying causative candidate gene *Glyma.20G85100*, encoding a CCT domain protein. Thus, efforts are needed to fine map or clone major QTLs controlling SPC in other grain legumes to delineate the underlying candidate gene(s) and their function for genomic-assisted breeding to improve SPC in grain legumes.

**Table 3:** List of seed protein content QTLs reported in various grain legumes.

Crop	Mapping Population/Panel of Genotypes	QTL/Gene	Marker	LG	PV%	References
Chickpea	GWAS, 187	4 QTLs, 9 significant MTAs	SSR	LG3, 5	2.4–5.1	[81]
	GWAS, 336	6 candidate genes	SNP	–	41	[170]
	ICC 995 × ICC5192, RIL (189)	*q-3.2*	SNP	LG3	44.3	[158]
Common bean	Xana × Cornell 49242, RIL (104)	*SpA*, *SpB*, *SpE*, *SpI*, *SpJ*, *Pha*, *SpF*, *SpG*, *SpK*, *SpL*, *SpM*, *SpC*, *SpD*	AFLP, RAPD, ISSR, SCAR	LG7, 4, 3, 1		[171]
	Xana × Cornell 49242, RIL (104)	One QTL	SSR	PV07	–	[172]
Ground nut	TG26 × GPBD 4, RIL (146)	8 QTLs	SSR	LG1, 3, 4, 7, 8	1.5–10.7	[173]
Pea	1238 × 1263, 110 × 807, 110 × 851 (F_2_)	Convicillin *(Cvc)*, Legumin *(Lg-1)*	Protein marker	LG2, 7	–	[145]
	360 × 611 (F_2_)	*Vicilin (Vc-1)*	–	LG7	–	[146]
	Wt10245 × Wt11238, F_2_ (114)	*prot1 prot2 prot3 prot4 prot5*	AFLP, RAPD, ISSR, STS, CAPS	LG2, 5, 7	13.1–25.5	[159]
	Térèse’ × K586, RIL (139)	14 QTLs			–	[119]
	Wt11238 × Wt3557 (F5), Wt10245 × Wt11238 (F5)	One QTL	AFLP, RAPD, STS, CAPS, ISSR	LGVa, 5b	–	[118]
	GWAS, 50	One significant SNP	SNP	–	–	[120]
	Orb × CDC Striker, Carrera × CDC Striker	8 QTLs	SNP	LG1b, 4a	16	[160]
	1–2347–144 × CDC Meadow			LG3b, 7b		
	GWAS, 135 genotypes	*Chr3LG5_194530376*	SNP		–	[174]
	GWAS, 135	*Chr3LG5_138253621*, *Chr3LG5_194530376*	SNP	LG3, 5	–	[174]
	9 populations, RIL (1213)	21 QTL	SNP	–	–	[123]
Pigeonpea	ICP11605 × ICP 14209, ICP 8863 × ICP 11605, HPL 24 × ICP 11605, ICP 8863 × ICPL 87119, ICP 5529 × ICP 11606	48 M-QTLs for SPC	SNP	CcLG03, 11, 02, 06	0.7–23.5	[157]
Soybean	Parker × PI 468916	Two major quantitative trait locus (QTL) alleles	–	LG20	–	[136]
	A3733 × PI 437088A, RIL (76)	One QTL	Satt496 and Satt239, RAPD marker OPAW13a	LG20	–	[175]
	Essex × Williams			LG6		[176]
	PI 97100 × Coker 237			LG15, 20		[177]
	N87-984-16 × TN93-99			LG18		[178]
	N87-984-16 × TN93-99, F_6_ (101)	4 QTL for cysteine, 3 QTL for methionine	Satt235, Satt252, Satt427, Satt436	D1a, F, G	–	[102]
			Satt252, Satt564, Satt590	F, G, M		
	A81356022 × PI 468916		–	LG20		[156]
	*G. soja* (PI468916) × *G. max* (A81-356022) backcrossing	One QTL	SSR, AFLP	LG1	–	[162]
	*G. max* A81-356022 × *G. soja* PI468916, near isogenic lines	One QTL, 13 genes	SNP	LG20		[162]
	Magellan × PI 438489B			LG15, 5, 6		[165]
	ZDD09454 × Yudou12			LG18, 20		[179]
	GWAS, 298	17 genomic regions, *Glyma20g19680*, *Glyma20g21030*, *Glyma20g21080*, *Glyma20g19620*, *Glyma20g196030*, *Glyma20g21040*	SNP	LG8, 9, 20	–	[180]
	IL-1964 (619 accessions), IL-1966 (977 accessions), MS- 1996 (728 accessions), MS-2000 (934 accessions)	–	SNP	LG20	–	[163]
	ZYD2738 × Jidou 12, F_2:3_, ZYD2738 × Jidou 9, F_2:3_	*qPRO_2_1*, *qPRO_13_1*, *qPRO_20_1*, *qPRO_6_1*, *qPRO_18_1*	SSR	LG2, 6, 13, 18, 20	6.6–14.5	[181]
	Benning × Danbaekkong	4 QTLs		LG14, 15, 17, 20	55	[132]
	R05-1415 × R05-638			LG14, 20		[182]
	GWAS, 139	*qPC19* and 8 significant genomic regions	SNP	LG5, 8, 10, 14, 16, 19	10.3	[183]
	SD02-4-59 × A02-381100 (RIL), SD02-911 × SD00-1501 (RIL)	8 QTLs	–	–	–	[184]
	Danbaekkong × *Glycine soja* (PI468916)	wp allele, cqSeed protein-003	–	LG20	–	[185]
	*G. max* (Williams 82) × *G. soja* (PI 483460B)	*5 QTLs: qPro_06*, *qPro_19*, *qPro_20*, *qPro_08*, *qPro_13*	SNP	LG6, 8, 13,19, 20	4.6–19.6	[167]
	GWAS, 144 lines derived from four parents	*Glyma.03G100800*, *Glyma.10G207300*, *Glyma.12G019300*, *Glyma.12G112900*, *Glyma.14G081600*, *Glyma.18G028600*, *Glyma.18G07110*, *Glyma.18G071300*	SNP	LG1, 2, 3, 4,6,7, 9, 10,12, 14, 18	3.84–19.21	[186]
	–	192 collinear protein QTLs, 13 candidate genes	–	–	–	[187]
	Linhefenqingdou × Meng 8206 RIL (104)	25 main effect QTLs	SNP	LG1, 4, 6, 7, 8, 9, 10, 13, 14, 17, 18, 19, 20	5.7–26.22	[169]
	GWAS, 621 accessions	Three genomic regions, 16 significant SNPs, *Glyma.15g049100*, *Glyma.15g049200*, *Glyma.15g050100*, *Glyma.15g050600*	SNP	LG4, 5, 8, 9, 10, 13, 15, 19, 20	–	[188]
	GWAS, 185	rs53140888, rs19485676, rs24787338	SNP	Chromosomes 1, 13, 20	–	[189]
		Three significant SNP markers				
	(Kenfeng14× Kenfeng15) × (Heinong48×Kenfeng19), RIL (160)	34 QTLs	SSR		2.65–13.83	[189]
	G15FN-12 mutant	–	SoySNP50K BeadChip	LG12	–	[190]
	GWAS, 249	25 significant MTAs	SNP	LG2, 6, 7, 10, 13, 14, 16, 17, 18, 19	–	[191]
	AC Proteusx Maple Arrow F_5_, RIL	5 QTLs	SSR, DArT and DArTseq	LG15, 20, 2, 18	70%	[168]
	X3145-B-B-3-15 × 9063, F_5_, RIL; X3145-B-B-3-15 × AC Brant, F_5_, RIL; X3144-48-1-B/9063, F_5_, RIL; X3144-48-1-B × AC Brant, F_5_, RIL; X3145-B-B-3-15 × X3144-48-1-B, F_5_, RIL			LG1, 8, 9, 14, 16, 17, 19, 20		
	AC X790P × S18-R6′ and ‘AC X790P × S23-T5, RILs	*qPro_Gm02–3*, *qPro_Gm04–4*, *qPro_Gm06–1*, *qPro_Gm06–3*, *qPro_Gm06–6*, *qPro_ Gm13–4*, *qPro-Gm15–3*	SNP	LG1, 2, 4, 5, 6, 8, 12, 13, 15, 18	10.4–21.9	[192]
	GWAS, 211	*qPC-7-1*, *qPC-13-1*, *qPC-15-1*	SNP	LG7, 13, 15	18–34	[164]
	(Kenfeng 14 × Kenfeng 15) × (Heinong 48 × Kenfeng 19)	85 QTL, 123 QTNs	2,232 SNPs and 63,306 SNPs	–	–	[193]
	G00-3213 × PI 594458A, 132 RIL	16 QTLs	SoySNP6k BeadChip	LG3, 6, 13, 20		[194]
	GWAS, 165	138 significant MTA,	SNP	LG7	–	[195]
		*Glyma.07g175700* and *Glyma.07g176000*				
	RIL, 944, Primus × Protina, Gallec × Sigalia, Primus × Sigalia, Protina × Sigalia, Gallec × Primus, Gallec × Protina, Sultana × Sigalia, Gallec × Protina, Gallec × Protina, Gallec × Sigalia, Primus × Sultana	*qPY1*, *qPY2*, *qPY3*, *qPY4*, *qPY5*, *qPY6*, *qPY7*	SNP	LG5, 6, 7, 8, 16, 18, 19, 20	15.5–60	[196]
	‘Nanxiadou 25′× Tongdou 11, RIL (178)	50QTLs, three candidate genes: *Glyma.20G088000*, *Glyma.20G111100*, *Glyma.20 g087600*	SNP	LG1, 2, 3, 5,6, 7, 8, 9, 10, 11, 13, 15, 16, 20	-	[197]
	PI 468916 × A81-356022, BC	*Glyma.20G85100*, *cqSeed protein-003* QTL	SNP	LG20	-	[137]
	250, F_2_	3 QTLs	Infinium Soy6KSNP Beadchips	LG6, LG13, LG20	-	[198]

AFLP = Amplified fragment length polymorphism; SNP = Single nucleotide polymorphism, SCAR = Sequenced cleaved amplified region, CAPS = cleaved amplified polymorphic sequence, RAPD = Random Amplified polymorphic DNA, SSR = Simple Sequence Repeats, ISSR = Inter Simple Sequence Repeat.

## 6. Underpinning Genomic Region/Haplotypes Controlling High Protein Content through GWAS

Traditional biparental QTL mapping for obtaining genetic recombinants controlling complex traits such as protein content is limited due to the incorporation of only two parents in the crossing program. However, the increased capacity of next generation sequencing technology to derive single nucleotide polymorphism molecular markers in association with advanced phenotyping facilities has facilitated the development of numerous genetic recombinants and identification of the underlying plausible candidate genomic regions controlling protein content in various grain legumes using GWAS [81,174,183,186]. Jadhav et al. [81] performed association mapping for SPC using SSR markers on a panel of 187 chickpea genotypes (desi, kabuli, and exotic). Nine significant marker trait associations (MTAs) for SPC were uncovered on LG1, LG2, LG3, LG4, and LG5, explaining 16.85% PV. A recent GWAS using high-throughput SNP markers on 140 chickpea genotypes subjected to drought and heat stress to shed light on MTAs with various nutrients uncovered 66 (non-stress), 46 (drought stress), and 15 (heat stress) MTAs for SPC [199], which could be used to identify high-protein lines for improving SPC in chickpea.

A GWAS relying on multilocation and multi-year phenotyping of a large set of pea germplasm representing diverse regions across the globe was undertaken to identify significant MTAs for agronomic and quality traits, including protein content [174]. Two significant MTAs controlling SPC were identified: Chr3LG5_138253621 and Chr3LG5_194530376.

GWAS using 16,376 SNPs in 332 chickpea genotypes (desi and kabuli) delineated seven genomic loci controlling SPC and explaining 41% combined PV [170]. The authors also validated five SPC-controlling genes in a RIL-based mapping population ICC 12299 × ICC 4958, encoding cytidine (CMP), deoxycytidylate (dCMP) deaminases, ATP-dependent RNA helicase DEAD-box, and zinc finger protein. An earlier comprehensive GWAS of 298 soybean lines using Illumina Infinium and GoldenGate assays identified 17 significant genomic regions controlling SPC [180]. Among the SPC-controlling genomic regions, LG20 was important as it contained six candidate genes *Glyma20g19680*, *Glyma20g21030*, *Glyma20g21080*, *Glyma20g19630*, *Glyma20g19620*, and *Glyma20g21040* in the 2.4 Mbp interval. Another GWAS performed on 139 soybean lines revealed eight significant regions contributing to SPC on LG5, LG8, LG10, LG14, LG16, LG19, and LG20 [183]. In addition, a major QTL *qPC19* controlling SPC on LG19 in the 42.3 to 44.2 Mb interval explained 10.3% PV [183]. Likewise, an assay using SoySNP660k BeadChip in 144 soybean lines developed from four-way RILs identified eight candidate genes controlling SPC: *Glyma.03G100800*, *Glyma.10G207300*, *Glyma.12G019300*, *Glyma.12G112900*, *Glyma.14G081600*, *Glyma.18G028600*, *Glyma.18G07110*, and *Glyma.18G071300* (Zhang et al. [186]; see Table 3). A comprehensive GWAS study in a collection of 877 soybean accessions, tested in five different environments in Midwest and southern USA using SoySNP50K iSelect BeadChip [188], identified significant genomic regions for SPC that coincided with previous QTL/genomic regions identified on chromosomes 15 and 20 [161,166]. Three SNPs identified within 91 kb overlapped the 118 kb genomic region of meta-QTL controlling SPC and seed oil content previously reported by Van and McHale [161]. Some important candidate genes identified in these genomic regions—*Glyma.15g049100 Glyma.15g049200*, *Glyma.15g050100*, and *Glyma.15g050600*—participate in partitioning carbon and regulating protein content (Lee et al. [188]; see Table 3). The authors also elucidated eight novel genomic regions controlling methionine, cysteine, lysine, and threonine contents. A GWAS using whole genome sequencing data of 631 soybean accessions combined with a biparental QTL analysis uncovered a pleotropic gene *GmSWEET39* (encoding sugar transporter) controlling SPC and seed oil content in soybean [164]. The authors also reported that a 2 bp (CC) deletion in *Glyma.15G049200* underlying the *GmSWEET39* allele rendered high seed oil content and low SPC.

A comprehensive association and linkage analysis surveyed 985 soybean accessions, including wild species, landraces, and old and modern cultivars, to capture haplotypic variation in the high SPC locus *cqProt-003* on chromosome 20 [200]. The study uncovered significant trait-associated genomic regions within a 173 kb linkage block containing three causal candidate genes: *Glyma.20G084500*, *Glyma.20G085250*, and *Glyma.20G085100* [200]. Of these, *Glyma.20G085100* (containing a 304 bp deletion and trinucleotide insertions) was tightly linked with the high protein content phenotype [200].

## 7. Functional Genomics Shedding Light on Causal Candidate Gene(s) Contributing Seed Protein Content in Grain Legumes

In the last decade, unprecedented advances in RNA sequencing have expedited functional genomics research, especially transcriptome analysis for discovering trait gene(s), in various grain legumes [197]. Numerous studies have elucidated various SPC-contributing candidate gene(s) and their functional roles in grain legumes; notably, cDNA cloning based functional characterization of genes encoding storage proteins such as pea seed albumin (PA1, PA1b) [201] and conglutin family in narrow leaf lupin [202]. Functional characterization of genes encoding storage protein in narrow leaf lupin by sequencing cDNA clones from developing seed identified 11 new storage protein (conglutin family)-encoding genes [202]. Transcriptome analysis via RNA-seq shed light on 16 conglutin genes encoding storage protein in the Tanjil cultivar of narrow leaf lupin [203]. Conglutin gene(s) expression is similar in lupin varieties of the same species but distinct between species [203]. In soybean, functional genomic analysis via gene expression profiling identified 329 differentially expressed genes underlying *qSPC_20–1* and *qSPC_20–2* QTL regions accounting for SPC using a QTL-seq approach [197]. Of the nine candidate genes underlying these QTL regions, *Glyma.20G088000*, *Glyma.20G111100*, and *Glyma.20 g087600* were functionally validated and identified as the most potential candidate genes controlling SPC [197]. RNAi technology—a robust functional genomic tool—offered novel insight into the regulatory role of *Glyma.20g085100* harboring transposon insertion in the SPC-controlling genomic region of soybean [137]. Reduced expression of *Glyma.20g085100* using RNAi enhanced the protein level in the low-protein Thorne soybean genotype [137]. Most functional genomics studies identifying SPC-controlling candidate genes with their putative function in major legumes have involved soybean; thus, studies should focus on elucidating candidate genes and deciphering the molecular mechanism for improving SPC via functional genomics in other grain legumes.

## 8. Proteomics and Metabolomics Shed Light on the Genetic Basis of High Seed Protein Content in Legumes

Proteomics helps us understand the entire set of proteins produced at a specific time under a particular set of conditions in an organism or cell [204]. This approach could be used to discover novel seed storage proteins and inquire about the molecular basis of enhancing SPC in various legumes [205]. A novel protein known as methionine-rich protein was discovered in soybean using a two-dimensional (2D) electrophoresis technique [205]. Later, a 2D-PAGE proteomic tool distinguished wild soybean (*G. soja*) from cultivated soybean based on high storage proteins (beta-conglycinin and glycinin) detecting 44 protein spots in wild soybean and 34 protein spots in cultivated soybean; thus, this helped in identifying high-protein soybean genotypes [206]. Combined SDS-PAGE and MALDI-TOF MS analysis in LG00-13260, PI 427138, and BARC-6 soybean genotypes revealed enhanced accumulation of beta-conglycinin and glycinins and thus high grain protein content compared to William 82 ([207]; see Table 4). A combined SDS-PAGE and MALDI-TOF MS analysis, comparing protein content in nine soybean accessions with William 82, revealed significant protein content differences in seed 11S storage globulins [208]. In common bean, proteome analysis of common bean deficient in seed storage proteins (phaseolin and lectins) revealed elevated sulfur amino acid content due to increased legumin, albumin 2, and defensin [209]. Santos et al. [210] characterized the protein content of 24 chickpea genotypes using a proteomics approach to explore genetic variability in storage protein. High-performance liquid chromatography analysis indicated the presence of sufficient genetic variability for SPC, with some genotypes rich in seven amino acids. In pea, a mature seed proteome map of a diverse set of 156 proteins identified novel storage proteins for enhanced SPC [211].

An iTRAQ-based proteomics analysis of CX (low SPC) and LX (high SPC) faba bean genotypes revealed differentially abundant proteins involved in amino acid metabolism [56]. Furthermore, a KEGG analysis suggested that valine, leucine, histidine, and β-alanine metabolism were significantly enriched by differentially abundant proteins [56].

Likewise, metabolomic studies help us understand various metabolic pathways and metabolites controlling protein accumulation during seed development [217]. A meticulous amino acid profiling study using contrasting high and low SPC soybean lines revealed that the ability of embryos to assimilate nitrogen and synthesize storage proteins determines SPC accumulation [217]. Further, the authors reported that high SPC at maturity is related to increased accumulation of asparagine in developing cotyledons.

**Figure 1:**
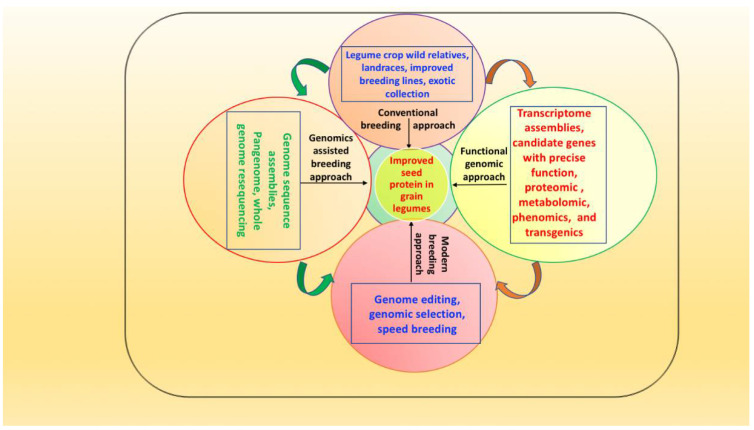
Integrated ‘omics’ and emerging novel breeding approach for improving protein content in grain legumes.

A metabolomics study using GC-TOF/MS in contrasting seed protein soybean lines showed a high abundance of metabolites (asparagine, aspartic acid, glutamic acid, free 3-cyanoalanine) that were positively associated with SPC and negatively associated with seed oil content [216]. However, various sugars (sucrose, fructose, glucose, mannose) had negative associations with seed protein and oil content [216]. Saboori-Robat et al. [218] undertook metabolite profiling of common bean genotypes differing in *S*-methylcysteine accumulation in seeds and found that *S*-methylcysteine accumulates as γ-glutamyl-*S*-methylcysteine during seed maturation, with a low accumulation of free methylcysteine. Amino acid profiling of Valle Agricola, a nutritionally rich chickpea genotype cultivated in southern Italy, revealed that 66% of the total amino acids comprised glutamic acid, glutamine, aspartic acid, phenyl alanine, asparagine, lysine, and leucine, while ~40% comprised histidine, valine, isoleucine, leucine, methionine and threonine [219]. Further advances in metabolomics could improve our understanding of various cellular metabolism networks and pathways related to SPC in legumes. Thus, integrating various ‘omics’ tools and emerging novel breeding approaches could assist in developing protein-fortified grain legumes (see Figure 1).

## 9. Progress of Genetic Engineering and Scope of Genome Editing for Improving SPC in Grain Legumes

Numerous studies have been undertaken to improve the essential amino acid content in various grain legumes by manipulating amino acid encoding genes using genetic engineering [220,221,222]. Many examples of improved essential amino acid contents, especially sulfur-rich amino acids, by manipulating gene(s) in various legumes using transgenic technology are available. Chiaiese et al. [223] introduced an albumin transgene encoding methionine and cysteine-rich protein from sunflower seed into chickpea to improve seed methionine content. The transgenic chickpea seed accumulated more methionine than the control. Likewise, Molvig et al. [224] improved seed methionine content in narrow leaf lupin by introducing sunflower seed albumin transgene at the transgenic level. However, cysteine-rich storage proteins, especially conglutin delta, declined in narrow leaf lupin seed due to low expression of the cysteine-encoding gene (Tabe and [225]; see Table 5). Introducing *Bertholletia excelsa* methionine-rich 2S albumin gene into common bean enhanced seed methionine content by more than 20% over non-transgenic plants [220]. Improving sulfur-rich amino acids, such as methionine and cysteine, in soybean has been a research priority, made possible by introducing the 15 kDa [226], 27 kDa [227], and 11 kDa [221,228] δ-zein encoding protein genes from maize using genetic engineering.

Despite some successes introducing transgenes to enhance SPC in grain legumes at the transgenic level, transgenic regulatory or governing bodies do not allow or restrict the use of these genetically engineered improved grain legumes commercially due to health and environmental safety issues. To overcome these stringent issues related to genetically modified crops, rapidly evolving genome editing technologies could help develop enhanced-protein grain legumes without introducing foreign genes. Using genome editing technologies, various crop plants have improved quality traits, such as increased fragrance and low gluten, starch, or oleic acid contents (for details, see [231]). However, the use of genome editing for SPC fortification in grain legumes is limited; future studies could adopt these powerful technologies to improve SPC by editing various gene(s), such as those encoding essential sulfur-rich amino acids or improving storage proteins.

## 10. Whole Genome Resequencing and Pangenome Sequencing for Elucidating Novel Structural Variants Related to High SPC across the Genome

Current breakthroughs in genome sequencing technologies have facilitated the sequencing of the global germplasm of various crops, including legumes, to underpin novel structural variants (SVs) such as presence/absence and copy number variations prevailing at the genome level [232,233]. An analysis combining association and biparental mapping using WGRS data of 631 soybean genotypes discovered a pleiotropic sugar transporter QTL gene *GmSWEET39* on chromosome 15 controlling SPC and seed oil content [164]. The authors suggested that deletion of 2 bp CC in the underlying causative *Glyma.15G049200* gene reduced SPC and enhanced seed oil content. Likewise, a pangenomic approach can describe the full complement of genes in the ‘core genome’ and ‘accessory genome’ to capture structural variation (not available in ‘single reference genome assembly’) at the species level [232]. Pangenome assemblies have been reported in chickpea [233], pigeon pea [234], soybean [235] and mungbean [236]. Thus, future construction and annotation of pangenomes for different grain legumes could reveal missing information on SPC structural variations in the available reference genome assemblies, expediting the development of grain legumes with enriched protein.

## 11. Non-Destructive Phenomics Approach for Quantifying High Protein Content in Grain Legumes

Several high-throughput phenotyping approaches have been developed to bridge the genotyping and phenotyping gap for various quality traits, including protein content [237,238,239]. Advances in high-throughput non-destructive phenotyping approaches such as hyperspectral technologies, near-infrared reflectance spectroscopy, and nuclear magnetic resonance have enabled the phenotyping of various biochemical attributes in cereal and legume seeds, including protein content, with high accuracy and efficiency [237,238,239,240,241]. For example, Raman spectroscopy has been used to measure SPC in soybean [237]. Earlier, near-infrared reflectance spectroscopy was used to screen high-protein soybean genotypes [242,243]. Thus, non-destructive high-throughput phenotyping approaches could save time when screening high-SPC lines.

## 12. Genomic Selection and Rapid Generation Advances for Selecting High SPC Lines to Increase Genetic Gain

Unprecedented advances in genome-wide molecular marker development allow the use of genomic selection (GS) for predicting the genetic merit of progenies with complex traits without observing their phenotypic values from large target populations by developing a prediction model and calculating genomic-assisted breeding values in a ‘training population’ with known phenotypic observation [244]. The benefit of GS for improving genetic gain could be harnessed by increasing selection intensity (i) and selection accuracy (I), and reducing the breeding cycle length (L) in the breeder’s equation: ΔG = R = h^2^S = σ_a_ × i × r/L. [ΔG = genetic gain, R = response to selection, h^2^ = heritability, σ_a_ = additive genetic variance]. Notable instances of using GS as a substitute for phenotypic selection for complex traits include grain yield under moisture stress in chickpea [245], common bean [246], cowpea (Ravelombola et al., 2021) [247], and pea [248,249] and cooking time in common bean [250]. However, GS has limited application for selecting high SPC genotypes in legumes [251]. A rrBLUP model was used to predict SPC in 306 pea genotypes derived from three RILs, tested in three autumn seasons in northern and central Italy, to determine any advantage of GS over phenotypic selection for SPC [251]. The mean predictive ability of GS for SPC was 0.53. Future studies could use GS to improve SPC and select various grain legume progenies with high SPC without phenotyping.

Likewise, the emerging benefits of speed breeding techniques could be harnessed by using optimum light intensity, photoperiod and temperature to enhance the rate of photosynthesis, resulting in early flowering and plant maturity, thus shortening the breeding cycle [252]. Speed breeding protocols have been established in chickpea, lupin, lentil, pea, soybean, and faba bean [253,254,255,256,257]. Further optimization of speed breeding protocols could fast-track improvements in various traits of breeding importance, including SPC, in grain legumes for sustaining global food security.

## 13. Fundamental Constraints on Seed Protein Content

As the offspring of plants, seeds are subject to several fundamental trade-offs that impact their size and composition. Seeds have fundamental required components, such as cell walls, and some amount of carbohydrates, lipids, and nucleic acids to make a viable embryo. Consequently, there are limits to potential selection on protein content. For example, long term selection on maize seed oil content has shown limits to the power of selection (e.g., [258]). Over the past two or more decades, ecologists have increasingly conceptualized these trade-offs as part of an economic spectrum, which influences the range of traits observed in leaves [259,260], stems [261,262] and roots [263]. As a dispersal unit, seeds are able to travel farther if they are smaller, but establish more readily if larger [264]. In many individual legume crops, wild relatives have presumably been under millenia of selection for these trade-offs in seed size and composition, limiting genetic variation and architecture. However, few researchers have linked evolutionary and ecological limits on seed composition to efforts at breeding, nor looked carefully at how they impact seed protein content. Seed size is generally an important co-variate in seed protein content, although among legumes its role differs somewhat among grain legumes.

Recent elegant work in chickpea suggests that these constraints are in fact real, and shape contemporary genetic diversity in seed size and composition. Chickpea has a QTL hotspot for seed size, leaf size, drought responses, and other “Vigour” traits. Nguyen and colleagues have recently fine mapped this QTL [265,266] showing it to be due to variation in a TIFY gene, which mutant studies in Arabidopsis have shown to impact seed size. Natural variation at this locus suggests it contributes significantly to a seed-size number trade-off, among parents that also differ in seed protein content.

## 14. Conclusions and Future Perspective

The increasing human population is facing increasing malnutrition-related problems such as dietary protein deficiency, especially in underprivileged and developing countries. Supplying protein-rich legumes improved through plant breeding and molecular breeding approaches could minimize the rising challenge of hunger and malnutrition-related problems. Moreover, improved grain legume dietary protein could be an important and economically viable alternative to high-cost animal-based dietary protein. Protein biofortification of major grain legumes will help satisfy the daily needs of human dietary protein in underprivileged and developing countries. Accurate characterization of various crop gene pool and landrace haplotypes with genetic variation for SPC needs urgent attention to accelerate SPC improvement in legumes. Harnessing the benefits of pre-breeding approaches could play a pivotal role in introgressing gene(s)/QTLs regulating high protein content from CWRs into high-yielding low-protein elite legume cultivars [96]. Recent advances in genomics, genome-wide association mapping, and whole genome resequencing approaches and the availability of complete genome and pangenome sequences in various legume crops could help underpin the causative alleles/QTLs/haplotypes/candidate genes controlling high protein at the genome level, enabling genomics-assisted selection for improving protein concentration in grain legumes. Likewise, functional genomics, proteomics, and metabolomics could enrich our understanding of the complex molecular networks controlling improved protein content in various grain legumes. Selecting protein-rich grain legume genotypes in assessed germplasm or segregating progenies is challenging as most protein-estimating processes are based on destructive methods. Thus, high-throughput non-destructive methods are important for selecting high-protein legume genotypes. Likewise, genomic selection and rapid generation advances could be important for selecting high-protein progenies and rapidly developing protein-dense legumes. To overcome the challenges of transgenic technology, genome editing will help us manipulate and edit genes(s) governing high protein content at specific locations on legume genomes to enhance SPC. Capitalizing on these modern breeding tools, we should be able to identify grain legumes with improved protein content without compromising yield, as these two traits have a strong inverse relationship [123]. Hence, the amalgamation of approaches could help combat the growing protein-based malnutrition and lower the hunger risk, ensuring sustainable human growth globally.

## Figures and Tables

**Table 1:** Seed protein contents and deficient amino acids in major grain legumes.

Crop		Scientific Name	Range of Grain Seed Protein Content	References	Deficient Amino Acids
Chickpea	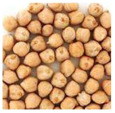	*Cicer erietinum* L.	17–22% before dehulling	[14,15]	Methionine, cysteine threonine and valine [16]
			25.3–28.9% after dehulling		
Lentil	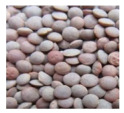	*Lens culinaris* Medik	20.6% and 31.4%	[17]	Methionine, cysteine [18]
Lupin	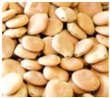	*Lupinus albus* L.	35–44%	[19,20]	Alanine, tryptophan [21]
Soybean	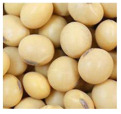	*Glycine max* (L.) Merr.	up to 40%	[22,23]	Methionine, cysteine, threonine and lysine [24]
Common bean	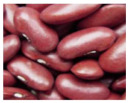	*Phaseolous vulgaris* L.	20–30%	[25,26]	Methionine, cysteine [27]
Pigeonpea	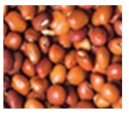	*Cajanus cajan* (L.) Millsp	20–22%	[28]	Methionine, cysteine, valine [29]
Faba bean	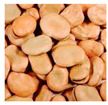	*Vicia faba* L.	26% to 41%	[30,31]	Methionine
Mung bean	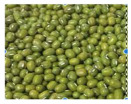	*Vigna radiata* L.	20.97–31.32%	[32]	Methionine, cysteine
Cowpea	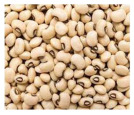	*Vigna unguiculata* L. Walp.)	14.8–25%	[33,34,35]	Methionine
Pea	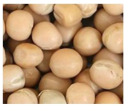	*Pisum sativum* L.	13.7 to 30.7%	[36,37,38]	Methionine, cysteine and tryptophan [39]
				[37,38,39]	
Urd bean	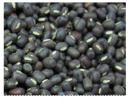	*Vigna mungo* L. Hepper	25–28%	[40,41]	Methionine, cysteine
Lathyrus	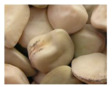	*Lathyrus sativus* L.	8.6–34.6%	[42]	Methionine, cysteine

**Table 2:** List of various legume genotypes with improved seed protein content.

Crop	Genotypes	Seed Protein Content	Source	References
Chickpea	ICC 5912	29.2%	ICRISAT, Patancheru, India	[78]
	LEGCA608, LEGCA609, LEGCA614, LEGCA619, LEGCA716	>22%	Cordoba	[84]
Common bean	J-216, FJIP-43	222 (J/L-146) to 330 (J-216); 180 (G11027A) to 311 (FJIP-43) g kg^−1^	Mexican state of Jalisco and Durango	[89]
	LR05	25.23%	Food Safety and Agricultural Research Center, Akdeniz University	[79]
	6-EX	23%	Santo Antônio de Goiás, Brazil	[90]
	Accession 4049	–	Portugal	[91]
Cowpea	HC-6, HC-5, CP-21, LST-II-C-12, CP-16, COVU-702, HC-98-64	26.7–27.9%	India	[86]
	TVu-2723, TVu-3638, TVu-2508	32.50%	Minjibir, Kano State, Nigeria	[77]
	MNC01-649F-2, BRS-Cauamé, BRS-Paraguaçu, BRS-Marataoã	27.4–28.3%	–	[88]
	“Early Scarlet” and 09-204	26.9–27.4%	Arkansas State (Fayetteville, Alma, Hope)	[92]
	Bengpla	40%	Dokpong and Bamahu near Wa, Ghana, South Africa, Taung	[93]
	PI662992, PI601085, PI255765, PI255774, PI666253	28.4–28.9%	Florida, Minnesota, Nigeria, Arkansas	[85]
	Vuli, Mamlaka, IT90K-59, Ngoji, TVU13953, 98K-5301		Tanzania, South Africa, Nigeria, South Africa, Nigeria	[34]
	Paulistinha	29.20%	Brazil	[87]
Faba bean	25 genotypes	28.43–29.68%	Manitoba and Saskatchewan, Canada	[94]
Grasspea	IC127616	32.20%	India	[95]
Lentil	*L. orientalis*	18.3–27.75%	India, IIPR, Kanpur	[96]
	*L. ervoides*	18.9–32.7%	India, IIPR, Kanpur	[96]
Mungbean	MGG330, Nagpuri	29.9% and 29.3%	India	[97]
Pea	PI206793, PI206801, PI206838, PI210619, PI210644, PI210675, PI210678, PI210684	>30%	Manitoba and Ontario, Canada	[98]
	Majoret	240.4 g kg^−1^	Grain Research Laboratory Winnipeg, Canada	[82]
	NGB 101293 Jordan	26.80%		[37]
	L1	317.63 g kg^−1^	Institute of Field and Vegetable Crops (Smederevska Palanka, Serbia)	[72]
Soybean	D76-8070	450 g kg^−1^	–	[99]
	BARC-6, BARC-7, BARC-8, BARC-9	–	–	[100]
	AC Proteus	–	Central Experimental Farm (Ottawa, ON) Canada	[101]
	TN03-350, TN04-5321	High protein content	Tennessee Agricultural Experiment Station, Tennessee, USA, USDA–ARS and the North Carolina Agricultural Research Service	[102]
	N6202′	–		[103]
	Lines developed from Kwangan- kong × Samnamkong and Danbaegkong × Samnam-kong	34.3–44.4% and 35.8–49.6%	Yeongnam Agricultural Research Institute (YARI), Milyang, Republic of Korea	[104]
	JIHJ117	53%	–	[105]
	17D derived population and M23 derived lines	382 and 403 g kg^−1^	University of Missouri Fisher Delta Research Center, Portageville, MO	[106]
	High-pro 1′ developed from Wyandot × GASF98-114	401 g kg^−1^	USDA Agricultural Service and Ohio Agricultural Research and Developmental Centre Wooster	[107]
	‘TN11-5102’	421 g kg^−1^ protein on a dry weight basis	University of Tennessee Agricultural Research	[108]
	PI407228	392.6–481.7 g kg^−1^	Central Crops Research Station in Clayton, NC, Bradford Farm in Columbia, Sandhills Research Station in Jackson Springs, NC	[109]
	R11-7999	439 g kg^−1^ (dry weight)	Arkansas Agricultural Experiment Station	[110]
	Bioagro	–	–	[111]
	S16-5540GT	41.10%	University of Missouri–Fisher Delta Research Center Soybean Breeding Program	[112]

**Table 4:** Proteomic approach for investigating novel proteins for improving seed protein content in grain legumes.

Crop	Protein Identified	Approach Used	Reference	Genotype
Chickpea	High amino acid content, 454 protein spots	Two-dimensional electrophoresis and mass spectrometry	[210]	Flip97-171C, Elite
Common bean	Sulfur-containing amino acids, S-methylcysteine accumulation	High resolution liquid chromatography-tandem mass spectrometry	[212]	–
	Sulfur-containing amino acids; enhanced concentration of cysteine and methionine	Mass spectrometry	[213]	SARC1 and SMARC1N-PN1
Faba bean	Amino acid metabolism	iTRAQ	[56]	Cixidabaican
	Legumin, vicilin, and convicilin	1D SDS-PAGE, size-exclusion high-performance liquid chromatography	[214]	Cartouche, NV657, NV734
Narrow-leafed lupin	2760 protein identifications	LC-MS	[215]	P27255, Tanjil, Unicrop
Pea	156 proteins	2-D gels, MALDI-TOF MS	[211]	Caméor
Soybean	High arginine content in Nepalese	MALDI-TOF; two-dimensional gel electrophoresis	[141]	Nepalese, Karve, Seti
	High beta-conglycinin and glycinins	Two-dimensional electrophoresis SDS-PAGE	[207]	LG00-13260
	High 11S storage globulins	SDS-PAGE, MALDI-TOF, two-dimensional electrophoresis	[208]	PI407788A
	High storage protein	2D-PAGE	[206]	Wild soybean
	Asparagine, free 3-cyanoalanine, and L-malic acid	GC-TOF/MS	[216]	

**Table 5:** Selected list of grain legumes with improved seed protein content using a genetic engineering approach.

Crop	Gene Source	Gene Name	Function	References	Transformation Approach
Chickpea	Sunflower	Sunflower seed albumin	Increased methionine up to 90%	[223]	*Agrobacterium tumefaciens*
Common bean	Brazilnut	Brazilnut 2S albumin	Increased methionine by 14–23%	[220]	Particle bombardment
Narrow-leafed lupin	Sunflower	Sunflower seed albumin	Increased methionine by 90%	[224,225]	*Agrobacterium tumefaciens*
	Arabidopsis	Serine acetyltransferase	26-fold increase in free cysteine	[229]	*Agrobacterium tumefaciens*
Soybean	Maize	15 kDa δ-zein	Increased methionine by 20% and cysteine by 35%	[226]	*Agrobacterium tumefaciens*
	Maize	27 kDa γ-zein	Increased methionine from 15.49 to 18.57% and cysteine from 26.97 to 29.33%	[227]	Particle bombardment
	Maize	11 kDa δ-zein	Methionine	[221]	*Agrobacterium tumefaciens*
		MB-16	Increased methionine by 16% and cysteine by 66%	[230]	Biolistic
	Soybean	Soybean plastid ATP sulfurylase isoform 1	Increase cysteine by 37–52% and methionine by 15–19%	[228]	*Agrobacterium tumefaciens*
	Maize	11 kDa δ-zein	Increased sulfur amino acids	[222]	*Agrobacterium tumefaciens*
	Soybean	*Glyma.20g085100*	Enhance protein content	[137]	RNAi technology

## Data Availability

The data presented in this study are available in the article.
